# Modeling of Information Diffusion in Twitter-Like Social Networks under Information Overload

**DOI:** 10.1155/2014/914907

**Published:** 2014-03-23

**Authors:** Pei Li, Wei Li, Hui Wang, Xin Zhang

**Affiliations:** College of Information Systems and Management, National University of Defense Technology, Changsha 410073, China

## Abstract

Due to the existence of information overload in social networks, it becomes increasingly difficult for users to find useful information according to their interests. This paper takes Twitter-like social networks into account and proposes models to characterize the process of information diffusion under information overload. Users are classified into different types according to their in-degrees and out-degrees, and user behaviors are generalized into two categories: generating and forwarding. View scope is introduced to model the user information-processing capability under information overload, and the average number of times a message appears in view scopes after it is generated by a given type user is adopted to characterize the information diffusion efficiency, which is calculated theoretically. To verify the accuracy of theoretical analysis results, we conduct simulations and provide the simulation results, which are consistent with the theoretical analysis results perfectly. These results are of importance to understand the diffusion dynamics in social networks, and this analysis framework can be extended to consider more realistic situations.

## 1. Introduction

Research on social networks has received remarkable attention in the past decade, since social networks provide numerous features to encourage information sharing among users. Among the existing social networks, microblogging services (e.g., Twitter and Facebook) have impressively become more and more popular, which provide new communication methods for people to stay connected with their friends. The use of microblogging for lightweight communication makes it important candidate media for informal communication.

Twitter is arguably one of the most well-known microblogging platforms currently available, which is used by hundreds of millions of people all over the world. Twitter users update their daily life activities by computers or mobile phones, so as to broadcast things that happen in their daily lives, such as what they are reading, thinking, and experiencing. Users declare the persons they are interested in by the action* following*. For the case when user *A* follows user *B*, we say user *A* is one of user *B*'s* followers*, and user *B* is one of user *A*'s* followees*. Twitter users are allowed to post short messages (up to 140 characters) which are so-called* tweets* and also forward messages which are so-called* retweets*. Each user has a web form, where all his/her followees' new messages (both tweets and retweets) are arranged in a reverse-chronological order. So after logging in, a user will get noticed if his/her followees have posted new messages.

Essentially, relationships in Twitter are asymmetric, since a user who is followed by another user does not necessarily have to reciprocate by following him/her back. Some social networks adopt symmetric relationships. For example, in Facebook, a relationship is established when a request for friendship is accepted by a user, which adds both on each other's contact lists. If one user removes the other, the relationship is broken. Therefore, an important difference between these two social networks is that the network of Twitter is directed, while that of Facebook is undirected. Having noticed the increasing popularity of Twitter, we take Twitter-like social networks into account in this paper.

Compared with traditional media such as newspapers and television, social networks allow creation and exchange of user-generated contents, while every user can produce and distribute messages. This results in an explosively growing amount of information and makes many social networks become increasingly information saturated. Besides, due to the potential for marketing and advertising, Twitter and some other social networks are considered to be efficient approaches to stimulate the awareness and adoption of products or services. One important benefit of these social networks is that the costs of generating and transmitting information are almost negligible, so advertising messages can reach wide audiences within a short period of time [1]. This also leads to a large volume of advertising information. However, due to the limitation of information-processing capability, if the messages arrive in numbers larger than what users can process, some messages will be lost without catching users' attention, where* information overload* occurs [2]. Under information overload, users will find it difficult to find useful messages according to their personal interests, which actually has a serious negative impact on the user experience. Therefore, to understand and then address the information overload issue arising in social networks, it is of importance to model and analyze the process of information diffusion under information overload, which is the focus of this paper.

Most research on diffusion dynamics in social networks has focused on the spread of one phenomenon at a time, for example, diffusion models for disease [3], influence [4], knowledge [5], and cooperation [6]. Recently, some researchers have begun to study competitive diffusion, which models the process that multiple competitive epidemics [7], influences [8], or phenomena [9] diffuse through a complex or social network. These problems are somewhat similar to the one considered in this paper, but they fail to characterize the information overload phenomenon in social networks, where every user can generate new messages. In our previous work [10, 11], we study the process of information diffusion under information overload in Facebook-like social networks. We know that the network structure of Twitter is very different from that of Facebook. Besides, considerable effort has been devoted to alleviate the information overload syndrome, where filter-based or cost-based approaches are usually adopted [12–14]. However, to the best of our knowledge, there is no prior work which seeks to model and analyze the process of information diffusion under information overload for Twitter-like social networks.

The remainder of this paper is organized as follows. We describe the models in Section 2 and analyze the process of information diffusion under information overload in Section 3. To verify the accuracy of theoretical analysis results, we conduct simulations and provide the simulation results in Section 4. Finally, we conclude this paper in Section 5.

## 2. Model Descriptions

In this section, we propose models to capture the characters of Twitter-like social networks, such as network, user behaviors, and information diffusion under information overload. Based on these models, we can analyze the process of information diffusion under information overload theoretically.

### 2.1. Network

**Figure 1:**
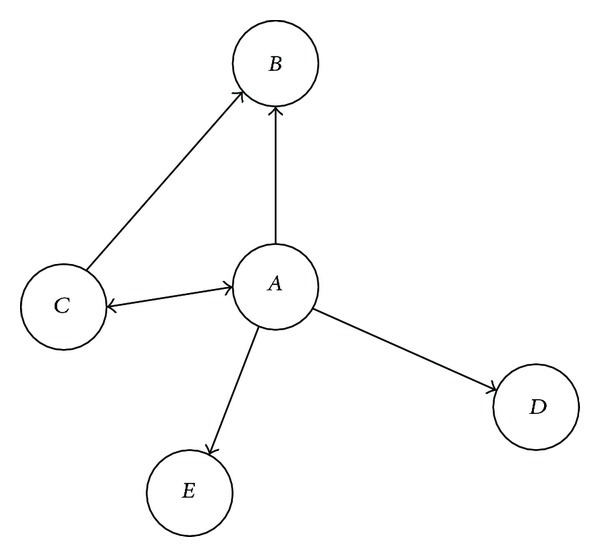
Network for Twitter-like social networks.

We consider Twitter-like social network as a directed network, where nodes represent typical users and links represent the relationships between pairs of users. Note that a user who is followed by another user does not necessarily have to reciprocate by following him/her back. We let the direction of a link be the same as the direction of information diffusion. For example, in Figure 1, user *A* is followed by users *B*, *C*, *D*, and *E*, where the update messages of user *A* can be received by users *B*, *C*, *D*, and *E*, and user *A* can only receive the update messages of user *C*.

Since isolated users never get involved in the process of information diffusion, we neglect all the isolated users and classify the rest of users into different types according to their in-degrees and out-degrees; that is, a user with in-degree *i* and out-degree *j* is of type (*i*, *j*), where *i* + *j* ≥ 1. For type (*i*, *j*) users, we define *e*_*k*,*l*_^*i*,*j*^ to be the probability that a randomly chosen follower is of type (*k*, *l*). Then, we have *j* ≥ 1, *k* ≥ 1, and

(1) $$\begin{array}{c} \sum_{k,l}e_{k,l}^{i,j}=1. \end{array}$$

We further define *q*_*i*,*j*_ to be the fraction of type (*i*, *j*) users in the network, and we get

(2) $$\begin{array}{c} \sum_{i,j}q_{i,j}=1. \end{array}$$

Consider the ensemble of networks in which the distributions {*e*_*k*,*l*_^*i*,*j*^} and {*q*_*i*,*j*_} take specified values. This defines a random graph model similar to the random graphs defined in [15, 16]. That is to say, the network is drawn uniformly at random from the ensemble of all possible networks with the distributions {*e*_*k*,*l*_^*i*,*j*^} and {*q*_*i*,*j*_}. For users, we denote by *M* the maximum number of in-degrees and by *N* the maximum number of out-degrees. Then, this network can be characterized by the (*M* + 1) × *N* × *M* × (*N* + 1) tensor {*e*_*k*,*l*_^*i*,*j*^} and the (*M* + 1)×(*N* + 1) matrix {*q*_*i*,*j*_}. Note that in a Twitter-like social network, users usually have moderate numbers of followees due to attention limitation. So, we usually have *M* ≪ *N*.

### 2.2. User Behaviors

In Twitter-like social networks, different functions are adopted to diffuse information. After logging in, users can post tweets to broadcast things which happen in their daily lives. There are also other functions such as reply and retweet which allow users to interact with their friends. In this paper, we generalize these behaviors into two categories:* generating* and* forwarding*; that is, users can generate new messages or forward messages generated by other users. Note that forwarded messages can still be forwarded.

To model the user ability of message processing under information overload, we introduce the term* view scope*, which indicates the messages a user can process at a time. Note that for users in Twitter-like social networks, messages are listed in a reverse-chronological order. So for a user with view scope number *S*, if information overload occurs, he/she can process (i.e., browse) the latest *S* messages after logging in, while the former ones are lost. In this paper, we assume homogeneous view scope number, which is *S*, for all users.

To model user behaviors, we make the same assumptions as [10].

- The process of user login follows a Poisson process with rate *λ*.
- After logging in and browsing the messages, a user may choose to log off or react to these messages (i.e., generate or forward a message), while the reacting probability is *p*_1_.
- Among the reacting actions, users may choose to forward a randomly chosen browsed message with probability *p*_2_ or generate a new message with probability 1 − *p*_2_.

Actually, user online activities may be bursty, and users may generate or forward multiple messages at a time. However, we make these assumptions to simplify the analysis here and plan to extend this analysis framework to more realistic situations in our future work.

### 2.3. Information Diffusion under Information Overload

**Figure 2:**
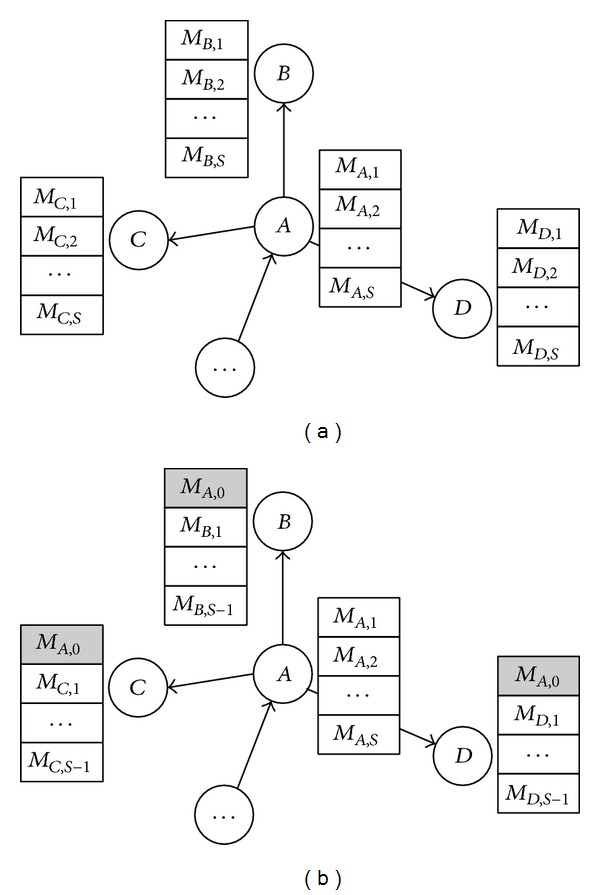
Evolvement of view scopes under information overload.

Under information overload, messages are arriving in numbers larger than what users can process, and some messages are lost without catching users' attention. We use Figure 2 to illustrate the evolvement of view scopes under information overload. Suppose user *A* is followed by other users, such as users *B*, *C*, and *D*. The view scopes of these users are depicted in Figure 2(a). After user *A* processes the messages in his/her view scope (i.e., *M*_*A*,1_, *M*_*A*,2_,…, and *M*_*A*,*S*_), he/she may generate a new message or just forward a message in his/her view scope. No matter which action is chosen, this message (say *M*_*A*,0_) will be placed at the top of all his/her followers' view scopes, and the messages at the bottom of his/her followers' view scopes (i.e., *M*_*B*,*S*_, *M*_*C*,*S*_, and *M*_*D*,*S*_) will be discarded due to the information overload effect, which are depicted in Figure 2(b).

One may argue that the view scope of user *A* should be cleared after he/she has processed all the messages. However, for simplicity we assume memoryless users here. That is to say, processed messages can still be processed as long as they are in the view scope. We will model the behaviors of users with memories in our future work.

## 3. Performance Analysis

In this section, we analyze the process of information diffusion under information overload based on the proposed models. Specifically, we are interested in the information diffusion efficiency, which is characterized by the average number of times a message appears in view scopes after it is generated by a type (*i*, *j*) user (say *u*_*i*,*j*_). To achieve this goal, we first calculate the average number of times a message is forwarded by a type (*k*, *l*) user after it arrives in this user's view scope (say *v*_*k*,*l*_).

### 3.1. Calculation of *v*_*k*,*l*_

Since users log in following a Poisson process with rate *λ*, we know that the probability that a user logs in and then generates or forwards a message within a time slot, which is of length Δ, is *λ*Δ*p*_1_. Consider a type (*k*, *l*) user (say user *B*). Note that he/she is memoryless and he/she may choose to forward a randomly chosen message in his/her view scope with probability *p*_2_ after he/she decides to react to the browsed messages. So if a message (say *M*_0_) is in his/her view scope, the average number of times that he/she will forward this message in *t* time slots is

(3) $$\begin{array}{c} f\left(t\right)=t\lambda \Delta p_{1}p_{2}\frac{1}{S}. \end{array}$$

The followees of user *B* will generate or forward messages, which will be placed at the top of his/her view scope. Let Δ → 0, and then, for user *B*, the probability that multiple followees generate or forward messages in the same time slot can be neglected. So the probability that a new message arrives in user *B*'s view scope in a time slot is *kλ*Δ*p*_1_. Note that message *M*_0_ will be discarded after *S* new messages arrive. Then the probability that message *M*_0_ will stay in user *B*'s view scope for *t* time slots is

(4) $$\begin{array}{c} g\left(t\right)=\left(\begin{array}{c} t-1\\ S-1 \end{array}\right){\left(1-k\lambda \Delta p_{1}\right)}^{t-S}{\left(k\lambda \Delta p_{1}\right)}^{S}. \end{array}$$

Therefore, the average number of times that user *B* will forward message *M*_0_ is

(5) vk,l=∑t≥0f(t)g(t)=∑t≥0tλΔp1p2S(t−1S−1)(1−kλΔp1)t−S(kλΔp1)S=λΔp1p2(kλΔp1)S∑t≥0(tS)(1−kλΔp1)t−S=λΔp1p2(kλΔp1)S∑t≥0(t+SS)(1−kλΔp1)t.

From (5.56) at [17, page 199], we get

(6) $$\begin{array}{c} \sum_{t\geq 0}\left(\begin{array}{c} t+S\\ S \end{array}\right)‍{\left(1-k\lambda \Delta p_{1}\right)}^{t}=\frac{1}{{\left(k\lambda \Delta p_{1}\right)}^{S+1}}. \end{array}$$

So, we have

(7) $$\begin{array}{c} v_{k,l}=\lambda \Delta p_{1}p_{2}{\left(k\lambda \Delta p_{1}\right)}^{S}\frac{1}{{\left(k\lambda \Delta p_{1}\right)}^{S+1}}=\frac{p_{2}}{k}. \end{array}$$

**Remark 1**

Intuitively, the larger the view scope number *S* is, the longer a message stays in the view scope and the more this message is forwarded. However, from (7) we find that *v*_*k*,*l*_ is unrelated to *S*. This is because larger *S* will lead to more messages stored in the view scope, which reduces the probability that a given message is chosen to be forwarded in a time slot.

### 3.2. Calculation of *u*_*i*,*j*_

Consider a type (*i*, *j*) user (say user *A*) and suppose his/her followers are divided into some partition {*r*_1,0_, *r*_1,1_,…, *r*_*M*,*N*_}, where *r*_*k*,*l*_ is the number of type (*k*, *l*) followers and

(8) $$\begin{array}{c} \sum_{k,l}r_{k,l}=j. \end{array}$$

The probability that the partition takes a particular value {*r*_*k*,*l*_} is given by the multinomial distribution [16]

(9) $$\begin{array}{c} P\left(j,\left\{r_{k,l}\right\}\right)=j!\prod_{k,l}\frac{1}{r_{k,l}!}{\left(e_{k,l}^{i,j}\right)}^{r_{k,l}}. \end{array}$$

We define the generating function *G*_*i*,*j*_(*z*) as the distribution of the number of times a message appears in view scopes after it is generated by a type (*i*, *j*) user and the generating function *H*_*k*,*l*_(*z*) as the distribution of the number of times a message appears in view scopes after it arrives in the view scope of a type (*k*, *l*) user. Then

(10) Gi,j(z)=∑{rk,l}δ(j,∑k,lrk,l)  ×P(j,{rk,l})H1,0(z)r1,0⋯HM,N(z)rM,N,

(11) $$\begin{array}{c} H_{k,l}\left(z\right)=zG_{k,l}{\left(z\right)}^{p_{2}/k}, \end{array}$$

where *δ* is the Kronecker delta function, and

(12) $$\begin{array}{c} \delta \left(j,\sum_{k,l}r_{k,l}\right)=\{\begin{array}{cc} 1, & \text{if}\;j=\sum_{k,l}r_{k,l},\\ 0, & \text{if}\;j\neq \sum_{k,l}r_{k,l}. \end{array} \end{array}$$

By submitting (11) into (10), we get

(13) Gi,j(z)=zj∑{rk,l}δ(j,∑k,lrk,l)×P(j,{rk,l})G1,0(z)p2r1,0⋯GM,N(z)p2rM,N/M.

Then, by submitting (9) into (13) and performing the sum over {*r*_*k*,*l*_}, we have

(14) $$\begin{array}{c} G_{i,j}\left(z\right)=z^{j}{\left(\sum_{k,l}e_{k,l}^{i,j}G_{k,l}{\left(z\right)}^{p_{2}/k}\right)}^{j}. \end{array}$$

By solving this equation, we can derive the distribution of the number of times a message appears in view scopes after it is generated by a type (*i*, *j*) user. However, here we just calculate the average number of times, which is

(15) $$\begin{array}{c} u_{i,j}=G_{i,j}^{\prime }\left(1\right)\;=j+jp_{2}\sum_{k,l}\frac{e_{k,l}^{i,j}}{k}G_{k,l}^{\prime }\left(1\right). \end{array}$$

We know that users with out-degree 0 can generate or forward messages, but no one can receive them. So, *G*_*i*,0_(*z*) = 1 and *G*_*i*,0_′(1) = 0. We further know that users with in-degree 0 never forward messages. That is to say, *G*_0,*j*_′(1) never contributes to the right of (15). So we can first calculate *G*_*i*,*j*_′(1) where *i*, *j* ≥ 1 and then get *G*_0,*j*_′(1) from (15).

We know that {*e*_*k*,*l*_^*i*,*j*^} becomes an *M* × *N* × *M* × *N* tensor for *i*, *j*, *k*, *l* ≥ 1, which is still hard to handle. We rearrange the elements of this tensor so that they form a matrix, which is called* matricizing* [18]. Specifically, we let

(16) $$\begin{array}{c} x=\left(i-1\right)N+j,\;\;y=\left(k-1\right)N+l. \end{array}$$

Then 1 ≤ *x*, *y* ≤ *MN* and

(17) $$\begin{array}{c} G_{x}^{\prime }\left(1\right)=j+jp_{2}\sum_{y}\frac{e_{x,y}}{k}G_{y}^{\prime }\left(1\right). \end{array}$$

We can write (17) in matrix form and get

(18) $$\begin{array}{c} G^{\prime }\left(1\right)=X1+p_{2}\;XEY{\;}^{-1}G^{\prime }\left(1\right), \end{array}$$

where

(19) 1=(1,1,…,1)T,X=(  X0  0⋯00  X0  ⋯0⋮⋮⋱⋮00⋯  X0  )M×M,X0=(10⋯002⋯0⋮⋮⋱⋮00⋯N),Y=(  Y1  0⋯00  Y2  ⋯0⋮⋮⋱⋮00⋯  YM  ),Ym=(m0⋯00m⋯0⋮⋮⋱⋮00⋯m)N×N.

So we get

(20) $$\begin{array}{c} \;G^{\prime }\left(1\right)={\left(I\;-p_{2}{XEY\;}^{-1}\right)}^{-1}X1\;. \end{array}$$

**Remark 2**

From (20), we observe that *u*_*i*,*j*_ is determined by *p*_2_ and {*e*_*k*,*l*_^*i*,*j*^} and is unrelated to other factors such as *S*, *p*_1_, and {*q*_*i*,*j*_}.

## 4. Simulations

To verify the accuracy of theoretical analysis results, we conduct simulations and provide the simulation results in this section. We first take into account a directed ER network and then a growing network model, which generates directed and degree-correlated networks.

The simulations are conducted in a discrete fashion. Specifically, time is slotted, and in each time slot a random user is selected to generate or forward a message. Denoting by *K* the user number, each simulation is run *KT* time slots, where *T* = 10000. That is to say, each user will be selected *T* times on average to generate or forward messages. We further set *S* = 10 and *p*_2_ = 0.5.

### 4.1. Directed ER Network

In the directed ER network, we let the user number *K* = 2001 and the average user in-degree (or out-degree) *α* = 10. That is to say, each link is included in the network with probability *p* = *α*/(*K* − 1) = 0.005.

**Figure 3:**
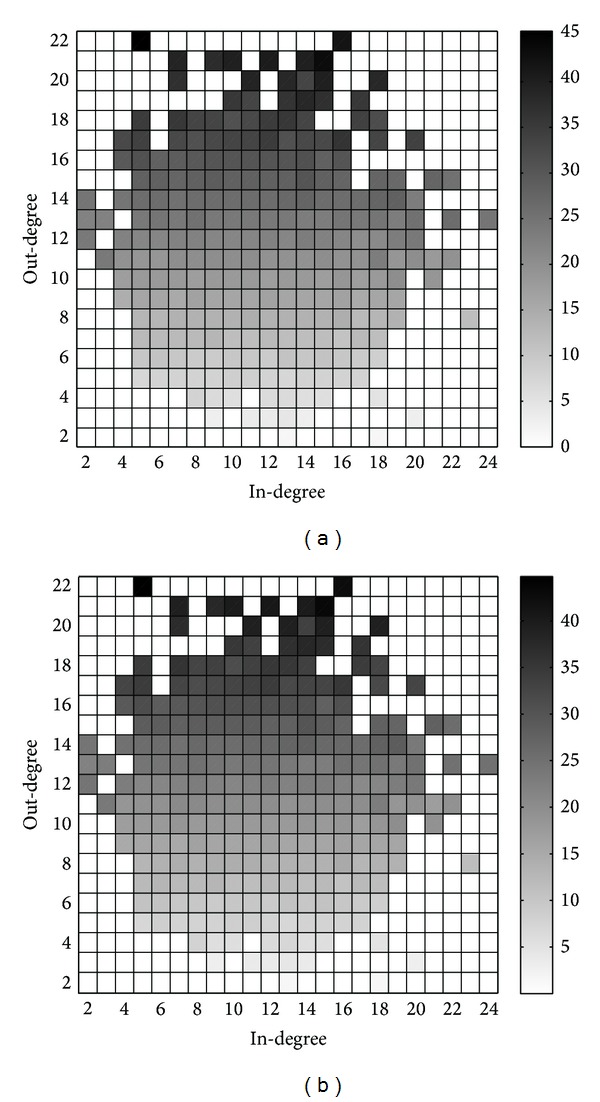
Results for *u*_*i*,*j*_ from (a) simulations and (b) theoretical analysis.

**Figure 4:**
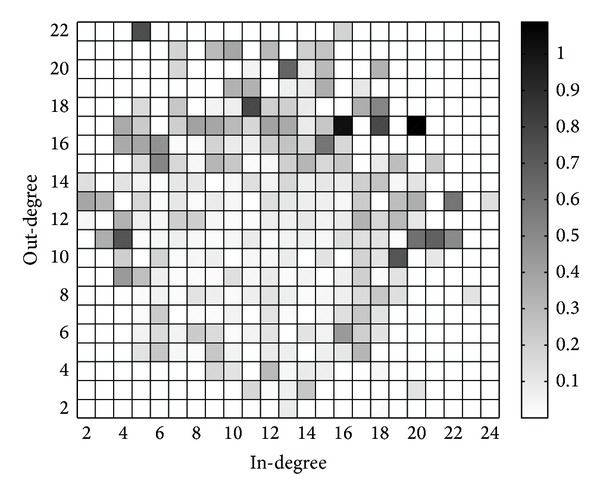
Differences between the results for *u*_*i*,*j*_ from simulations and theoretical analysis.

The results for *u*_*i*,*j*_ from simulations and theoretical analysis (i.e., (20)) are depicted in Figure 3. To quantify the gap between these results, we plot the differences in Figure 4, from which we know that the theoretical analysis results coincide very well with the simulation results.

### 4.2. Growing Network Model

Degree correlations among nodes in a network essentially characterize the network structure, while many real-world networks show degree correlations [19–21]. In particular, social networks show assortative mixing, that is, a preference of high-degree nodes to be connected to other high-degree nodes [15, 16].

To generate directed and degree-correlated networks, we adopt the growing network model proposed in [22], where in each step the probability of adding a new node and creating a link from one of the earlier nodes (say *A*) is

(21) $$\begin{array}{c} q\frac{d_{A}^{\text{out}}+\beta }{\sum_{C\in V}\left(d_{C}^{\text{out}}+\beta \right)}, \end{array}$$

and the probability of adding a new link and connecting two old nonlinked nodes (say from *A* to *B*) is

(22) $$\begin{array}{c} \left(1-q\right)\frac{d_{A}^{\text{out}}+\beta }{\sum_{C\in V}\left(d_{C}^{\text{out}}+\beta \right)}\frac{d_{B}^{\text{in}}+\gamma }{\sum_{C\in V}\left(d_{C}^{\text{in}}+\gamma \right)}, \end{array}$$

where *V* is the node set, *d*_∗_^out^ (*d*_∗_^in^) is the out-degree (in-degree) of node ∗, and parameters *β*, *γ* must obey the constraints *β* > 0 and *γ* > −1 to ensure that each node will be chosen with positive probability.

**Figure 5:**
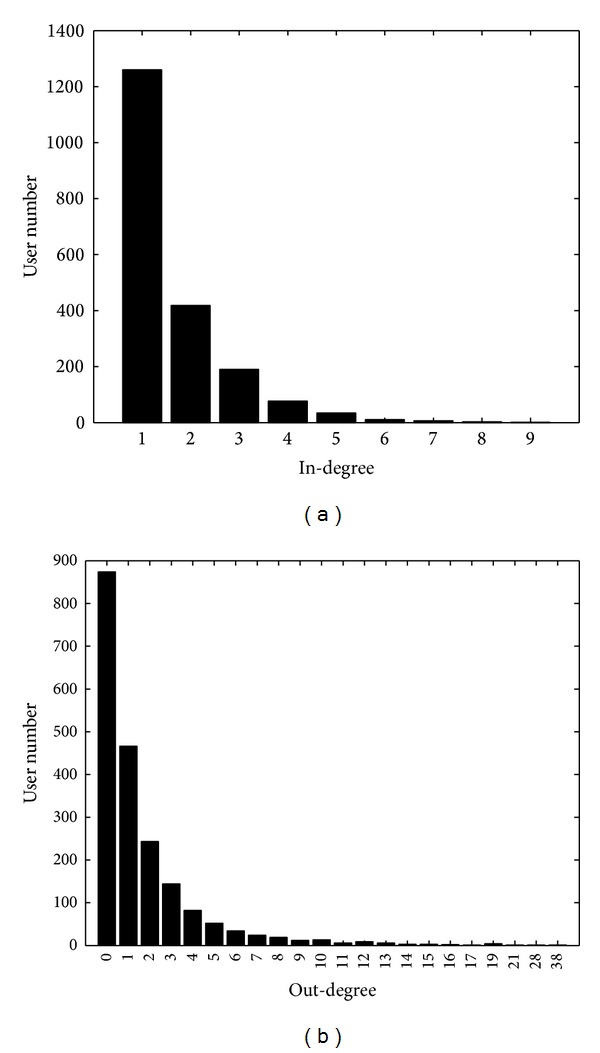
Distributions of (a) in-degrees and (b) out-degrees.

**Figure 6:**
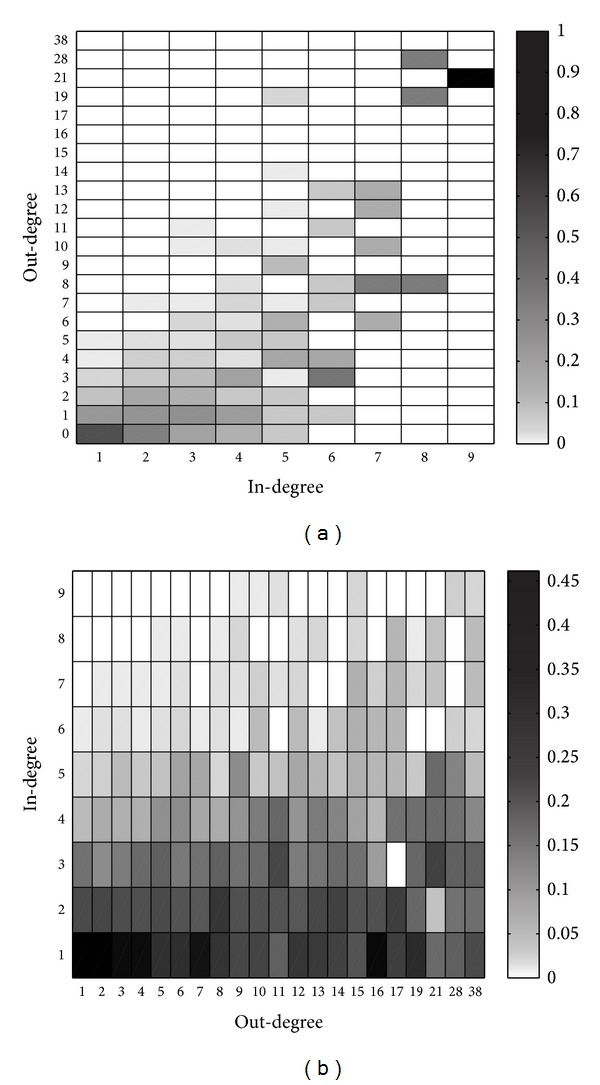
Degree correlations. (a) Degree correlations at users. Columns indicate the distributions of out-degrees for users with given in-degrees. (b) Degree correlations at links. Columns indicate the distributions of followers' in-degrees for users with given out-degrees.

Here, we set *K* = 2000, *q* = 0.6, and *β* = *γ* = 5 to generate a degree-correlated network. The distributions of in-degrees and out-degrees are depicted in Figure 5, from which we know that in-degrees and out-degrees follow power law distributions. The degree correlations are depicted in Figure 6, from which we know that degree correlations at users are evident, while high in-degree users usually have high out-degrees. However, the degree correlations at links are not so obvious.

**Figure 7:**
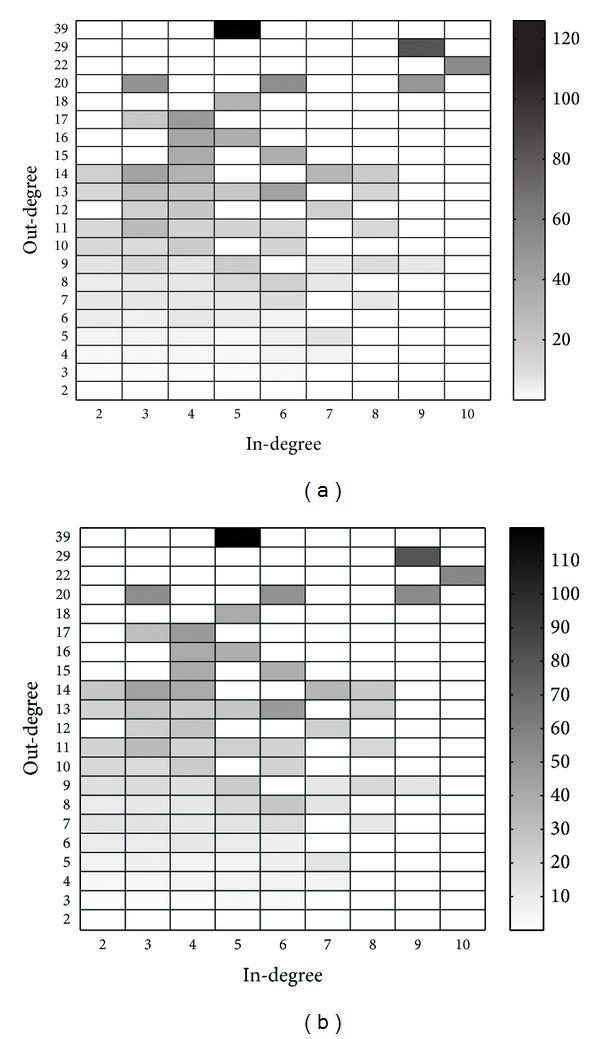
Results for *u*_*i*,*j*_ from (a) simulations and (b) theoretical analysis.

**Figure 8:**
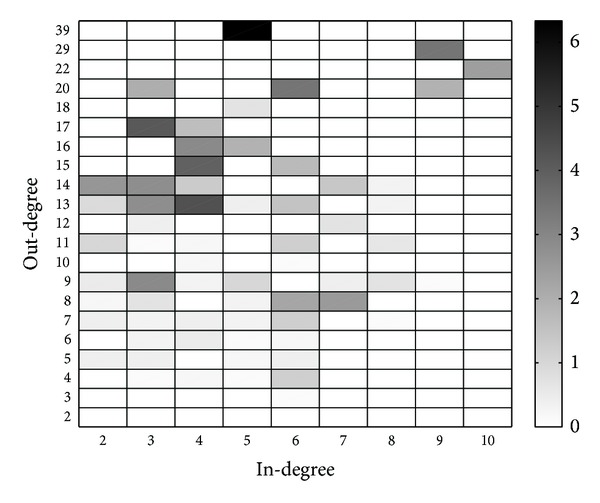
Differences between the results for *u*_*i*,*j*_ from simulations and theoretical analysis.

Simulation results for *u*_*i*,*j*_ are depicted in Figure 7(a), while the theoretical analysis results are depicted in Figure 7(b). We also plot the differences in Figure 8, from which we know that the theoretical analysis results are quite consistent with the simulation results, especially for users with low in-degrees and out-degrees. However, even for the only user who is of type (5,39), the value of difference is about 6, which is very small compared to the value of *u*_5,39_.

## 5. Conclusion

Having noticed the increasing popularity of Twitter and negative influence of information overload, we take Twitter-like social networks into account and propose models to capture the characters such as network, user behaviors, and information diffusion under information overload. Based on these models, we analyze the process of information diffusion under information overload theoretically, and the accuracy of theoretical analysis results is verified by simulations. These results are of importance to understand the diffusion dynamics in social networks and of use for advertisers in viral marketing. However, to simplify the analysis, we make some assumptions such as Poisson arrival and memoryless users, which seem to be unrealistic. We seek to extend these models to characterize more realistic situations and validate the theoretical analysis results by empirical evidence in our future work. Besides, the impact of degree correlations on spreading dynamics appears to be nontrivial [23], and it is demonstrated that degree correlations strongly influence information diffusion [24]. Another future work of this paper is to analyze the impact of degree correlations on the information diffusion under information overload.
